# Effect supplementation of black soldier fly larvae oil (*Hermetia illucens* L.) calcium salt on performance, blood biochemical profile, carcass characteristic, meat quality, and gene expression in fat metabolism broilers

**DOI:** 10.1016/j.psj.2023.102984

**Published:** 2023-08-01

**Authors:** Muhammad Anang Aprianto, Muhlisin, Asih Kurniawati, Chusnul Hanim, Bambang Ariyadi, Muhsin Al Anas

**Affiliations:** ⁎ Department of Animal Nutrition and Feed Science, Faculty of Animal Science, Universitas Gadjah Mada, Sleman, Yogyakarta, Indonesia; † Department of Animal Production, Faculty of Animal Science, Universitas Gadjah Mada, Sleman, Yogyakarta, Indonesia

**Keywords:** black soldier fly larvae oil, calcium salt, performance, meat quality, gene expression

## Abstract

This study evaluated the effect supplementation of black soldier fly larvae oil calcium salt (**BSFLO-SCa**) on performance, blood biochemical profile, carcass characteristic, meat quality, and gene expression in fat metabolism broiler chickens. A total of 280 male New Lohmann strain MB 202 broiler chicks (1-day-old) were randomly placed into 4 treatments, including a control group (T0) were fed basal diet and a basal diet supplemented with 1% (T1), 2% (T2), and 3% (T3) BSFLO-SCa. Each treatment consisted of 7 pens with 10 chickens each. Results showed that 1% BSFLO-SCa supplementation significantly reduced (*P* < 0.05) abdominal and meat fat, while gene expression on fat synthesis (FAS, ACC) was downregulated. Meat fatty acid profiles such as medium-chain fatty acid being dominant in lauric and myristic and monosaturated fatty acid significantly increased (*P* < 0.05). On the other hand, polyunsaturated fatty acid significantly decreased (*P* < 0.05). In addition, the other parameters did not affect by supplementation of 1% BSFLO-SCa. The addition starting from 2% significantly reduced (*P* < 0.05) performance and carcass characteristics. Blood biochemical profiles (HDL, protein, albumin) and meat qualities (protein, cholesterol, water-holding capacity, cooking losses, a* (redness), and b* (yellowness) values) were significantly increased (*P* < 0.05), while gene expression on fat oxidation (CPT-1) was upregulated. In conclusion, broiler chicken that received of 1% BSFL-SCa does not negatively affect growth performance and carcass characteristics but reduced fattening in broiler meat.

## INTRODUCTION

Chicken meat is the available and affordable primary source of high-quality protein for the world's community. Broiler chicken is a fast-growing poultry commodity whose production is projected to continue to increase along with the demand for chicken meat which is driven by the population growth rate which continues to increase (Saminathan et al., 2022). Feed energy values are needed to meet fast-growing chicken requirements, so their cost and demand increase (Benzertiha et al., 2019). The inclusion of oil in the diet is a method to increase dietary energy density due to the high caloric value produced (Ravindran et al., 2016; Attia et al., 2020). Moreover, the oil improves feed palatability, absorption of fat-soluble vitamins, and absorption of feed nutrients by reducing the feed flow rate (Baião and Lara, 2005; Poorghasemi et al., 2015). Besides, fat and fatty acids composition in muscle reflects dietary fatty acid composition, which affects the meat quality and nutritional value (Aghwan et al., 2014; Abdulla et al., 2015; Khatun et al., 2018).

Crude palm oil (**CPO**) is widely used in the poultry industry because relatively cheap with high energy content. Moreover, CPO contains high saturated fatty acid (**SFA**) (46%), mainly palmitic acid (Wan Nooraida and Abidah, 2020). The high content of SFA in palm oil increase in serum total cholesterol, LDL-cholesterol, and HDL-cholesterol, which triggers coronary artery disease (**CAD**) (Sun et al., 2015; Hinrichsen, 2016), so it has become a consideration of consumers at this time. In the current study, many researchers focus on utilizing black soldier fly larvae oil (**BSFLO**) resulting from the BSF larvae extraction process in animal feed, especially in the poultry industry (Wang and Shelomi, 2017). The fatty acid content in BSFLO is high with medium-chain fatty acid (**MCFA**), mainly lauric acid (C12:0), until 52% (Ewald et al., 2020).

MCFA is the most effective energy source because it easily and quickly undergoes an oxidation process in the liver (Dabbou et al., 2021). The energy source is rarely deposited in the subcutaneous fat tissue so carcasses with a relatively low-fat content are produced. The utilization of BSFLO has been reported to decrease meat fat and cholesterol (Cullere et al., 2019; Dabbou et al., 2021) and abdominal fat (Nilugonda et al., 2022). Fat deposition in muscle is a balance between lipolysis and lipogenesis in the body, which is regulated by changes in gene expression involved in fat metabolism. All the genes involved are influenced by nutrition which will determine the process of fat metabolism (Dev et al., 2021). On the other hand, the problem with using liquid fat in developing countries is the need for proper facilities for mixing with solid feedstuff. Calcium salt form of fatty acids is a saponification process that may alleviate this problem, easy to handle (Abd-ElhayGado and Elsebaai, 2017; Shahryari et al., 2021), higher stability and resistant to oxidation (Çalik et al., 2019; Villanueva-Lopez et al., 2020).

Many studies have aimed to determine the effect of BSFLO on blood lipid profiles and meat quality in broilers. However, the research results are inconsistent. To the best of our knowledge, there are no studies related to mRNA expression on fat metabolism in broiler livers effect of BSFLO. Therefore, in this study, we evaluate the effect of replacement CPO with BSFLO-calcium salt on performance, carcass characteristic, blood biochemical profile, meat quality, and lipid metabolism gene expression in broiler.

## MATERIALS AND METHODS

All animal procedures performed in this study were registered with the Research Ethics Committee at the Faculty of Veterinary Medicine, Universitas Gadjah Mada No. 00149T/EC-FKH/Ex./2021.

### Preparation of Black Soldier Fly Larvae Oil Calcium Salt

Black soldier fly larvae oil (**BSFLO**) in this study was obtained from the PT. Magalarva Sayana Indonesia (Banten, Indonesia). The BSFL-calcium salt (**SCa**) was prepared by mixed BSFL oil with NaOH solution and vigorously stirred until solid. Then, CaCl_2_ solution was added and vigorously stirred until was obtained yellowish solid particles. The BSFLO and BSFLO-SCa measured the fatty acid profile using gas chromatography (**GC**; Agilent Technologies 7890B, California) according to the method presented by Mjøs (2003) with modifications. The chromatogram peaks can be identified based on the retention time and compared with commercial standards. The fatty acid compositions of BSFLO and BSFLO-SCa are presented in Table 1.

**Table 1:** Fatty acid profiles of black soldier fly oil larvae (BSFLO) and black soldier fly larvae oil calcium salt (BSFLO-SCa).

Profil Asam Lemak	BSFLO	BSFL-Sca
Decanoic (C10:0)	1.13	0.79
Lauric (C12:0)	38.96	30.38
Myristic (C14:0)	8.53	8.34
Pentadecanoic (C15:0)	0.15	0.19
Palmitic (C16:0)	17.41	21.02
Heptadecanoid (C17:0)	0.14	0.17
Stearic (C18:0)	2.56	nd
Heneicosanoic (C21:0)	nd	0.15
Docosanoic (C22:0)	nd	nd
Lignoceric (C24:0)	nd	0.16
Palmitoleic (C16:1)	2.27	2.99
Oleic (C18:1)	16.48	0.16
Linoleic (C18:2)	9.16	30.62
Gamma-linolenic (C18:3)	nd	0.16
Eicosanoic (C20:1)	1.04	0.70
Linolenic (C18:3)	0.90	0.91
Eicosatrienoic (C20:3)	0.18	0.10
Eicosatetranoic (C20:1)	0.21	nd
Nervoic (C24:1)	0.20	0.15
SFA	68.88	61.20
MUFA	20.20	4.00
PUFA	10.24	31.79

Abbreviations: MUFA, monounsaturated fatty acids; PUFA, polyunsaturated fatty acids; SFA, saturated fatty acids.

### Animals and Housing

A total of 280-day-old male chicks of the New Lohmann Indian River (MB 202 Platinum) that had been vaccinated against Newcastle disease (**ND**) and Gumboro (infectious bursal disease) from the hatchery were put into brooding cages for 10 d. Chickens were weighed on d 11 and randomly housed with an initial body weight (**BW**) of 350 ± 10 g in 28 colony cages with a size of 1 × 0.75 m, 10 birds per cage. Each cage is equipped with a place to feed and drink. The broiler management followed the recommendations of the Indian River broiler management handbook (Aviagen, 2018). The maintained room temperature was 30°C until 3 d and then reduced to 2.5°C per wk until a temperature of 20°C was achieved. Lighting programs provide for a long day with 23 h of light and 1 h of darkness in the early stage of growth up to 7 d. After 7 d of age, around 5 h of darkness may be optimum (4–6 h).

### Experimental Treatments and Design

This study used a 1-way pattern design (completely randomized design). Each treatment consisted of 7 replicates, and each replicate contained 10 birds. The birds in each group were fed a basal diet based on crude palm oil as a control (T0) and substituted with 1% (T1), 2% (T2), and 3% (T3) of BSFLO-SCa. Feeding trials were given from the age of 11 d to 35 d. The starter phase (1–10 d) used commercial feed, and the grower phase (11–21 d) and the finisher phase (22–35 d) were formulated according to Aviagen (2022) recommendations, as is shown in Table 2. Water and feed are freely available ad libitum. The fatty acid compositions of the experimental diets as is shown in Table 3.

**Table 2:** Compositions and nutrient content of experimental grower (11–21 d) and finisher (22–35 d) diets.

	Percentage (%)							
	Grower (11–21 d)				Finisher (22–35 d)			
Feed ingredients	0%	1%	2%	3%	0%	1%	2%	3%
Corn	50.00	51.44	52.85	54.30	56.42	57.85	59.27	60.37
Rice bran	6.49	5.10	3.70	2.25	5.90	4.48	3.07	2.00
Soybean meal	34.00	34.00	34.00	34.00	28.50	28.50	28.50	28.50
Meat bone meal	3.00	3.00	3.00	3.30	3.00	3.00	3.00	3.00
Crude palm oil	3.00	2.00	1.00	0.00	3.00	2.00	1.00	0.00
BSFLO-SCa	0.00	1.00	2.00	3.00	0.00	1.00	2.00	3.00
Limestone	1.20	1.15	1.13	1.07	1.07	1.06	1.00	0.98
Dicalcium phosphate	0.88	0.88	0.89	0.95	0.76	0.76	0.81	0.80
NaCl	0.33	0.33	0.33	0.33	0.32	0.32	0.32	0.32
Vitamin mix^1^	0.04	0.04	0.04	0.04	0.04	0.04	0.04	0.04
Mineral mix^2^	0.30	0.30	0.30	0.30	0.30	0.30	0.30	0.30
DL-methionine	0.20	0.20	0.20	0.20	0.17	0.17	0.17	0.17
L-lysine	0.22	0.22	0.22	0.22	0.20	0.20	0.20	0.20
L-threonine	0.05	0.05	0.05	0.05	0.03	0.03	0.03	0.03
Choline chloride	0.09	0.09	0.09	0.09	0.09	0.09	0.09	0.09
Toxin binder	0.20	0.20	0.20	0.20	0.20	0.20	0.20	0.20
Nutrient content								
Crude protein (%)	21.60	21.55	21.50	21.44	19.75	19.70	19.64	19.61
Ether extract (%)	5.95	5.47	4.99	4.51	5.99	5.51	5.03	4.57
Crude fiber (%)	4.03	3.91	3.79	3.66	3.96	3.84	3.72	3.62
ME (kcal/kg)	3169	3166	3162	3158	3226	3222	3218	3212
Ca (%)	0.87	0.87	0.87	0.87	0.79	0.79	0.79	0.79
Available P (%)	0.44	0.44	0.44	0.44	0.40	0.40	0.40	0.40
Methionine (%)	0.53	0.53	0.52	0.52	0.47	0.47	0.47	0.47
Lysine (%)	1.36	1.35	1.35	1.34	1.20	1.20	1.19	1.19
Threonine (%)	0.89	0.89	0.88	0.88	0.80	0.79	0.79	0.79

1 Supplied per kg if diet: vitamin A, 50,000,000 IU; vitamin D3, 10,000,0000 IU; vitamin E, 80,000 mg; vitamin K3, 10,000 mg; vitamin B1, 10,000 mg; vitamin B2, 30,000 mg; vitamin B3, 225,000 mg; vitamin B5, 62,000 mg; vitamin B6, 10,000 mg; vitamin B9, 5,000 mg; vitamin B12, 100 mg; vitamin H, 100 mg; vitamin C, 20,000 mg.

2 Supplied per kg of diet: Mn, 40,000 mg; Fe, 32,000 mg; Cu, 6,050 mg; Zn, 32,000 mg; I, 404 mg; Se, 100 mg.

**Table 3:** Fatty acid compositions of experimental diets.

	Percentage (%)							
	Grower				Finisher			
Fatty acids	0%	1%	2%	3%	0%	1%	2%	3%
Decanoic (C10:0)	nd	0.21	0.21	0.56	nd	0.18	0.26	0.38
Lauric (C12:0)	0.52	8.77	12.34	17.50	0.67	5.13	10.42	14.33
Myristic (C14:0)	0.84	2.97	3.84	5.00	0.85	1.98	3.15	4.17
Heptadecanoid (C17:0)	0.29	0.88	0.87	1.44	0.27	0.57	0.95	1.25
Stearic (C18:0)	nd	nd	nd	0.11	nd	nd	nd	0.12
Heneicosanoic (C21:0)	nd	0.20	0.29	0.30	0.28	0.24	0.19	0.23
Tricosanoic (C23:0)	nd	0.11	0.19	0.11	0.20	0.18	0.16	0.14
Lignoceric (C24:0)	nd	nd	nd	nd	nd	nd	nd	0.11
Palmitoleic (C16:1)	32.26	28.62	25.66	22.08	30.60	29.68	25.33	20.25
Oleic (C18:1)	4.08	3.99	4.02	4.01	3.97	4.11	3.83	3.68
Linoleic (C18:2)	34.16	29.78	26.51	25.28	33.45	29.94	26.82	23.14
Gamma-linolenic (C18:3)	23.94	21.81	21.32	20.51	26.87	24.96	26.25	28.31
Eicosanoic (C20:1)	0.54	0.42	0.17	0.34	0.51	0.46	0.14	0.35
Linolenic (C18:3)	1.19	1.30	1.44	1.40	1.20	1.22	1.46	1.73
Nervoic (C24:1)	nd	nd	nd	nd	nd	0.19	nd	nd
SFA	1.65	13.14	17.74	25.02	2.27	8.28	15.13	20.73
MUFA	36.88	33.03	29.85	26.43	35,08	34.44	29.30	24.28
PUFA	59.29	52.89	49.27	47.19	61.52	56.12	54.53	53.18

Abbreviations: MUFA, monounsaturated fatty acids; PUFA, polyunsaturated fatty acids; SFA, saturated fatty acids.

### Growth Performance and Carcass Characteristics

On 21 and 35 d, all birds and the remaining feed in each cage were weighed to determine the final BW, body weight gain (**BWG**), feed intake (**FI**), and feed conversion ratio (**FCR**). Index performance (**IP**) was calculated at the end of the experiment. On d 35, a total of 56 birds (14 birds per treatment with 2 birds per replicate) with BW close to the median for each group were selected, weighed, and slaughtered by decapitation and cutting the jugular vein with halal method at commercial slaughter-house for carcass assessments. After feathering, evisceration, and the neck, head, and feet were removed manually from each carcass, abdominal fat was collected and weighed. The hot carcasses were subsequently placed in ice water (at 4°C) for approximately 4-h chill in ice box and transported to the laboratory. Carcass, breast, and thigh were weighed to analyze their yield. The yield percentages of carcass and abdominal fat were calculated relative to live weight, while the yield percentages of breast and thigh were calculated relative to cold carcass.

### Blood Profiles

At the end of the experiment (d 35), blood serum samples were collected from 28 birds (7 birds per treatment with 1 bird per replicate) with a BW close to the median for each group was selected, weighed and slaughtered by decapitation and cutting the jugular vein, for which separated blood serum samples were collected in Eppendorf tubes and preserved at a very low temperature of −20°C until analyzed. The total protein, albumin, glucose, total cholesterol, HDL-cholesterol, LDL-cholesterol, and triglyceride concentration were determined using a UV-visual photometer (Microlab 200: Merck Vital Scientific, Darmslandt, Netherlands) suitable with the commercial kits (DiaSys Diagnostic System GmbH, Holzheim, Germany).

### Meat Quality Measurements

The birds that have been slaughtered for blood sampling then feathering, evisceration, and cleaning. The breast meat was taken to measure for chemical quality, including water, ash, protein, fat (AOAC, 2005), and cholesterol content using the Liebermann-Burchard method (Shafiq et al., 2022). Physical quality, including water-holding capacity (**WHC**), cooking loss, and meat tenderness measured according to the procedure (Khan et al., 2021). The meat colors were determined by colorimeter (CR-400, Minolta Camera Co., Osaka, Japan) and were set to the L* (lightness), a* (redness), and b* (yellowness) after 45 min postmortem with a 65 light source and a 2°C observer (Bai et al., 2022a).

### Breast Meat Fatty Acid Profiles

The lipid was extracted by acid hydrolysis method from approximately 5 g of breast meat sample using 10 mL 3 M aqueous HCl at 80°C for 3 h and petroleum ether (Soxhlet extraction). Then, the extract was methylated using 1.0 mL of methylation reagent, which consisted of 75% of 2.5 M methanolic HCl and 25% of toluene (Xiao et al., 2012). After methylation, the fatty acids were quantified as methyl esters (**FAME**) using GC (Agilent Technologies 7890B, California), equipped with a 100 × 0.3 m BPX-70 cyanopropyl column with 0.2 uM film thickness (SGE, Ringwood, Victoria, Australia). Helium has used a mobile phase under the pressure of 2.20 bar. The injector temperature was 260°C. The oven was programmed as follows: 100°C for 5 min and 240°C for 10 min. The sample solution (1.0 uL) was injected splitless, and the split was opened after 2 min. The FAMEs were identified by comparing the elution pattern and relative retention time with reference FAME mixture (GLC-793, Nu-Chek Prep Inc., Elysian, MN) (Mjøs, 2003).

### Lipid Metabolism Gene Expression in Quantitative Real-Time PCR

Liver samples from 1 bird in each replicate (28 birds) were taken and collected in a microtube. Microtubes were frozen in liquid nitrogen immediately and stored at −80°C until analyzed. Gene expression analysis begins with RNA extraction from the liver sample of as much as 20 mg using a Quick-RNA miniprep kit (Zymo Research Corp., Irvine, California) according to procedures. The RNA purity and quantity were measured using Nanodrop Spectrophotometer (Maestrogen Inc., Hsinchu City, Taiwan). The total RNA was used as a template for cDNA synthesis with reverse transcriptase enzyme using ReverTrace qPCR RT Master Mix (Toyobo Co., Ltd., Osaka, Japan, Cat No. FSQ-301). Relative gene expressions were performed using QuantStudio 3 Real-Time PCR system (Thermo Fisher Scientific, Waltham, MA) and Thunderbird SYBR qPCR Mix (Toyobo Co., Ltd., Osaka, Japan, Cat No. QPX-201) according to the procedure. Briefly, 2 uL diluted cDNA, 6 pmol forward primer, 6 pmol reverse primer, 0.04 uL ROX reference dye, and 10 uL qPCR Mix were filled to the tube and placed into a 20 uL reaction volume with nuclease-free water. All primer pairs used for fatty acid synthase (**FAS**), acetyl-Coa carboxylase (**ACC**), carnitine palmitoyltransferase 1 (**CPT-1**), and 3-hydroxy-3-methylglutaryl coenzyme A reductase (**HMGR**) gene expression can be found in Table 4. The following amplification program was used: a hold stage for 1 cycle of 95°C for 2 min, a PCR stage for 40 cycles of 95°C for 1 s and 60°C for 30 s. The melt curve was analyzed at the end of the run to determine specific product amplification. There were 7 samples for each group, and each sample was performed in duplicate. The mRNA levels were standardized as the ratio to b-actin in arbitrary units by the 2^−ΔΔCT^ method and the data were expressed as the relative values to the control group (Livak and Schmittgen, 2001).

**Table 4:** Primer pairs for analysis of fat metabolism gene expression.

Gen	Primer sequence (5′−>3′)	Orientation	Base pairs	References
β-actin	GTGTGATGGTTGGTATGGGC	Forward	225	Xie et al. (2019)
	CTCTGTTGGCTTTGGGGTTC	Reverse		
FAS	TGGTTGACTGCCACCAATTG	Forward	213	Xie et al. (2019)
	ACCCCACTTTCCATCACGAT	Reverse		
HMGR	TCCCTGAACCCTCATCTTTG	Forward	250	König et al. (2007)
	TCTGCAAGAATACGGCTCCT	Reverse		
ACC	GCTGGGTTGAGCGACTAATG	Forward	173	Xie et al. (2019)
	GGGAAACTGGCAAAGGACTG	Reverse		
CPT-1	GAAGACGGACACTGCAAAGG	Forward	223	Xie et al. (2019)
	GGGCAAGTTGAATGAAGGCA	Reverse		

Abbreviations: ACC, acetyl-Coa carboxylase; CPT-1, carnitine palmitoyltransferase; FAS, fatty acid synthase; HMGR, 3-hydroxy-3-methylglutaryl coenzyme A reductase.

### Statistical Analyses

All experimental data were analyzed statistically using IBM SPSS statistic version 26.0. The data subjected to 1-way ANOVA among 4 treatments. A Duncan test was used to determine significant differences among all treatments. The statistical significance of all analyses was set at *P* < 0.05 for probability values.

## RESULTS

### Growth Performance

The effects of dietary supplementation with BSFLO-Sca on growth performance are presented in Table 5. During the interval of 11 to 21 d, dietary supplementation with 2 and 3% BSFLO-Sca significantly decreased (*P* < 0.05) FBW and BWG. During the interval of 22 to 35 d and 11 to 35 d, dietary supplementation with 2 and 3% BSFLO-Sca significantly decreased (*P* < 0.05) FI, FBW, BWG, and IP, while significantly increased FCR (*P* < 0.05). Overall, dietary supplementation with 1% BSFLO-SCa did not affect performance parameters of broiler.

**Table 5:** Broiler performance with dietary supplementation of black soldier fly larvae oil calcium salt (BSFLO-SCa).

	Treatments					
Parameters	0%	1%	2%	3%	SEM	*P* value
11–21 d						
FI (g)	899.64	869.14	857.64	849.64	9.75	0.755
FBW (g)	986.28^a^	965.14^ab^	954.14^b^	949.14^b^	4.85	0.024
BWG (g)	635.57^a^	614.21^ab^	603.35^b^	597.71^b^	4.88	0.022
FCR	1.41	1.41	1.42	1.42	0.01	0.730
IP	332.20	324.90	319.87	320.02	3.42	0.120
22–35 d						
FI (g)	1835.71^a^	1771.69^ab^	1723.42^b^	1610.82^c^	19.86	<0.001
FBW (g)	2049.78^a^	2013.52^a^	1888.21^b^	1881.92^b^	19.56	<0.001
BWG (g)	1063.50^a^	1048.38^a^	934.07^b^	932.78^b^	16.92	0.001
FCR	1.68^b^	1.69^b^	1.77^a^	1.72^ab^	0.01	0.040
IP	347.49^a^	330.02^a^	303.75^b^	299.29^b^	5.30	0.001
11–35 d						
FI (g)	2735.35^a^	2640.84^ab^	2581.07^b^	2460.46^c^	24.74	<0.001
FBW (g)	2049.78^a^	2013.52^a^	1888.21^b^	1881.92^b^	19.56	<0.001
BWG (g)	1699.07^a^	1651.44^a^	1537.42^b^	1512.38^b^	19.98	<0.001
FCR	1.59^c^	1.60^bc^	1.64^a^	1.63^ab^	0.00	0.003
IP	363.99^a^	349.14^ab^	327.28^bc^	316.83^c^	5.55	<0.001

a–c Means within a column with different superscripts are different (*P* < 0.05). Abbreviations: BW, body weight; BWG, body weight gain; FCR, feed conversion ratio; FI, feed intake; IP, index performance; SEM, standard error of the mean.

### Carcass Characteristics

Carcass characteristics due to effect of dietary supplementation with BSFLO-Sca are presented in Table 6. Dietary supplementation with 2 and 3% significantly decreased (*P* < 0.05) slaughter weight, carcass weight, breast weight, and thigh weight. However, supplementation with 1% BSFLO-SCa did not affect carcass characteristics in broiler. Abdominal fat was significantly decreased (*P* < 0.05) by a diet supplemented with BSFLO-Sca.

**Table 6:** Effect supplementation of black soldier fly larvae oil calcium salt (BSFLO-SCa) on broiler slaughter weight, carcass, and abdominal fat (*n* = 14 per treatment).

	Treatments					
Parameters	0%	1%	2%	3%	SEM	*P* value
Slaughter weight (g)	2209.00^a^	2182.08^a^	2093.33^ab^	2105.00^b^	18.067	0.018
Carcass yield (g)	1540.90^a^	1518.41^ab^	1454.83^b^	1468.80^b^	12.292	0.023
Carcass percentage (%)	69.73	69.50	69.50	69.77	0.200	0.537
Breast yield (g)	552.09^a^	549.84^ab^	501.72^b^	502.13^b^	6.777	0.001
Breast percentage (%)	34.40	33.96	34.28	34.80	0.242	0.694
Thigh yield (g)	494.50^a^	487.23^a^	460.66^ab^	449.64^b^	6.365	0.028
Thigh percentage (%)	31.42	31.67	31.89	31.22	0.146	0.418
Abdominal fat (g)	22.31^a^	18.46^b^	15.81^c^	13.49^d^	0.751	<0.001
Abdominal fat (%)	1.03^a^	0.86^b^	0.76^c^	0.67^d^	0.033	<0.001

a–d Means within a column with different superscripts are different (*P* < 0.05). Abbreviation: SEM, standard error of the mean.

### Blood Profiles

The effects of dietary supplementation with BSFLO-Sca on blood profiles are presented in Table 7. Dietary supplementation with 3% significantly increased (*P* < 0.05) HDL-cholesterol, while 2% had no significant difference from the control and 1% had the lowest HDL-cholesterol. A significant difference (*P* < 0.05) in dietary treatment effects was also noted for total protein and albumin, which were higher at 2 and 3% compared to the other treatment groups. There was no significant difference (*P* > 0.05) on triglyceride, total cholesterol, LDL-cholesterol, and glucose among treatments.

**Table 7:** Blood lipid profile of broilers fed with black soldier fly larvae oil calcium salt (BSFLO-SCa) at 35 d of age (*n* = 7 treatment).

	Treatments					
Parameter	0%	1%	2%	3%	SEM	*P* value
Triglyceride (mg/dL)	46.43	46.01	46.69	48.03	0.675	0.762
Cholesterol (mg/dL)	138.01	134.92	143.47	141.23	2.068	0.508
HDL (mg/dL)	43.09^b^	30.27^c^	42.77^b^	61.06^a^	2.443	0.000
LDL (mg/dL)	66.33	58.00	67.56	66.17	2.098	0.366
Glucose (mg/dL)	137.68	135.54	146.85	140.97	3.316	0.670
Protein (g/dL)	2.08^b^	2.31^b^	3.04^a^	2.83^a^	0.111	0.003
Albumin (g/dL)	1.54^b^	1.55^b^	1.97^a^	1.77^ab^	0.056	0.009

a–c Means within a column with different superscripts are different (*P* < 0.05). Abbreviations: HDL, high-density lipoprotein; LDL, low-density lipoprotein; SEM, standard error of the mean.

### Meat Quality

The effects of dietary supplementation with BSFLO-Sca on meat quality are presented in Table 8. Dietary supplementation had an effect in meat chemical quality which significantly decreased (*P* < 0.05) in fat while 2 and 3% significantly decreased (*P* < 0.05) cholesterol content and significantly increased (*P* < 0.05) in protein. Meat physical quality also influenced in dietary treatments effects which 2 and 3% significantly increased (*P* < 0.05) in WHC while 3% significantly decreased (*P* < 0.05) in a cooking loss. A significant difference (*P* < 0.05) was noted for a* and b* which values were higher for 2 and 3% of dietary treatments.

**Table 8:** Effect supplementation of BSFLO-SCa on the chemical and physical quality of broiler meat aged 35 d (*n* = 7 per treatment).

	Treatments					
Parameters	0%	1%	2%	3%	SEM	*P* value
Chemistry						
Water (%)	74.81	74.47	74.90	74.11	0.132	0.129
Ash (%)	5.41	5.19	5.00	5.16	0.078	0.184
Fat (%)	5.69^a^	4.79^b^	3.96^c^	4.54^b^	0.151	<0.001
Protein (%)	21.63^b^	21.70^b^	22.15^a^	22.89^a^	0.134	<0.001
Cholesterol (mg/100 g)	794.69^a^	771.44^a^	685.77^b^	685.43^b^	12.319	<0.001
Physical						
WHC (%)	29.54^b^	35.33^ab^	36.43^a^	39.67^a^	1.191	0.015
Cooking loss (%)	27.13^a^	26.48^a^	26.38^a^	24.60^b^	0.319	0.023
Shear force (kg/cm^2^)	1.70	1.77	1.73	1.75	0.060	0.981
Meat color						
L*	56.75	55.38	55.30	54.16	0.571	0.484
a*	1.94^b^	2.23^b^	3.40^a^	2.98^a^	0.139	<0.001
b*	7.61^c^	8.26^bc^	9.67^ab^	10.34^a^	0.337	0.008

a–c Means within a column with different superscripts are different (*P* < 0.05). Abbreviations: a*, redness; b*, yellowness; L*, lightness; SEM, standard error of the mean; WHC, water-holding capacity.

### Breast Meat Fatty Acid Composition

Fatty acid composition of breast meat in broiler chickens highly affected with dietary supplementation of BSFLO-SCa (Table 9). A significant difference (*P* < 0.05) was noted in most saturated fatty acid profiles which values increased such as lauric (C12:0) and myristic (C14:0). Different from most SFA, palmitic acid (C16:0) decreased with the increasing the BSFLO-SCa supplementation. As a result, the total SFA significantly increased (*P* < 0.05) with dietary supplementation of BSFLO-SCa.

**Table 9:** Fatty acid profile of broiler meat with the supplementation of black soldier fly larvae oil calcium salt (BSFLO-SCa) at 35 d of age (*n* = 7 per treatment).

	Treatments					
Parameter	0%	1%	2%	3%	SEM	*P* value
Lauric (C12:0)	0.35^d^	1.37^c^	2.12^b^	3.04^a^	0.199	<0.001
Myristic (C14:0)	0.89^d^	1.36^c^	1.84^b^	2.34^a^	0.109	<0.001
Pentadecanoic (C15:0)	0.19	0.21	0.21	0.25	0.009	0.115
Palmitic (C16:0)	24.45^b^	25.70^a^	22.73^c^	22.65^c^	0.295	<0.001
Heptadecanoid (C17:0)	3.62^a^	3.66^a^	2.87^b^	2.97^b^	0.109	0.006
Heneicosanoic (C21:0)	0.40^ab^	0.45^a^	0.35^b^	0.32^b^	0.014	0.008
Docosanoic (C22:0)	0.28^b^	0.33^b^	0.42^a^	0.46^a^	0.017	<0.001
Tricosanoic (C23:0)	3.33^b^	3.84^b^	4.90^a^	4.18^ab^	0.187	0.018
Lignoceric (C24:0)	0.11^b^	0.11^b^	0.18^ab^	0.20^a^	0.015	0.047
Pentadecanoic (C15:1)	0.18^a^	0.04^b^	0.04^b^	0.65^b^	0.015	<0.001
Palmitoleic (C16:1)	0.58	0.58	0.57	0.54	0.012	0.599
Heptadecanoic (C17:1)	0.10^ab^	0.12^a^	0.06^b^	0.00^c^	0.012	<0.001
Oleic (C18:1 ω9)	6.81^a^	7.26^a^	8.51^b^	8.74^b^	0.197	<0.001
Linoleic (C18:2 ω6)	36.83^a^	35.00^a^	32.04^b^	32.29^b^	0.499	<0.001
Gamma-linolenic (C18:3 ω6)	18.66	18.49	18.53	18.85	0.184	0.916
Eicosanoic (C20:1 ω6)	0.66^b^	0.68^b^	0.69^b^	0.75^a^	0.011	0.041
Linolenic (C18:3 ω3)	0.22	0.20	0.21	0.21	0.003	0.069
Eicosatrienoic (C20:3 ω3)	0.99	0.93	0.92	0.96	0.033	0.866
Erucic (C22:1 ω9)	0.04	0.04	0.07	0.09	0.011	0.211
Eicosatetranoic (C20:1 ω3)	0.13^b^	0.39^a^	0.18^a^	0.17^a^	0.029	0.006
Nervoic (C24:1 ω3)	0.76^a^	0.30^b^	0.03^c^	0.04^c^	0.059	<0.001
Docosahexaenoic (C22:6 ω3)	0.33	0.25	0.53	0.43	0.030	0.002
SFA	33.73^b^	37.04^a^	35.70^a^	36.56^a^	0.335	<0.001
MUFA	9.29^b^	9.43^b^	10.17^a^	10.42^a^	0.150	0.009
PUFA	56.7^a^	54.63^ab^	51.70^c^	52.32^bc^	0.553	0.001
PUFA/SFA	1.69^a^	1.48^b^	1.46^b^	1.44^b^	0.025	<0.001
n6	56.16^a^	54.18^ab^	51.26^c^	51.90^bc^	0.569	0.003
n3	2.45^a^	2.07^b^	1.89^b^	1.83^b^	0.071	0.004
n6/n3	23.05^b^	26.45^b^	28.21^ab^	29.72^a^	1.102	0.044

a–d Means within a column with different superscripts are different (*P* < 0.05). Abbreviations: MUFA, monounsaturated fatty acids; n3, Omega-3 fatty acids; n6, Omega-6 fatty acids; PUFA, polyunsaturated fatty acids; SEM, standard error of the mean; SFA, saturated fatty acids.

On the other hand, the monounsaturated fatty acid (**MUFA**) evidenced an increase with supplementation of BSFLO-SCa (*P* < 0.05) such as oleic (C18:1 ω9), eicosanoic (C20:1 ω6), and eicosatetranoic (C20:1 ω3) which determined increased of total MUFA in broiler breast meat. On the contrary, the polyunsaturated fatty acid (**PUFA**) was decreased with supplementation of BSFLO (*P* < 0.05). The decreased of PUFA was mainly due to the reduced of linoleic (C18:2 ω6) with supplementation of BSFLO (*P* < 0.05). Consequently, the dietary supplementation of BSFLO-SCa significantly decreased PUFA/SFA and increased n-6/n-3 ratio (*P* < 0.05).

### Lipid Metabolism Gene Expression

**Figure 1:**
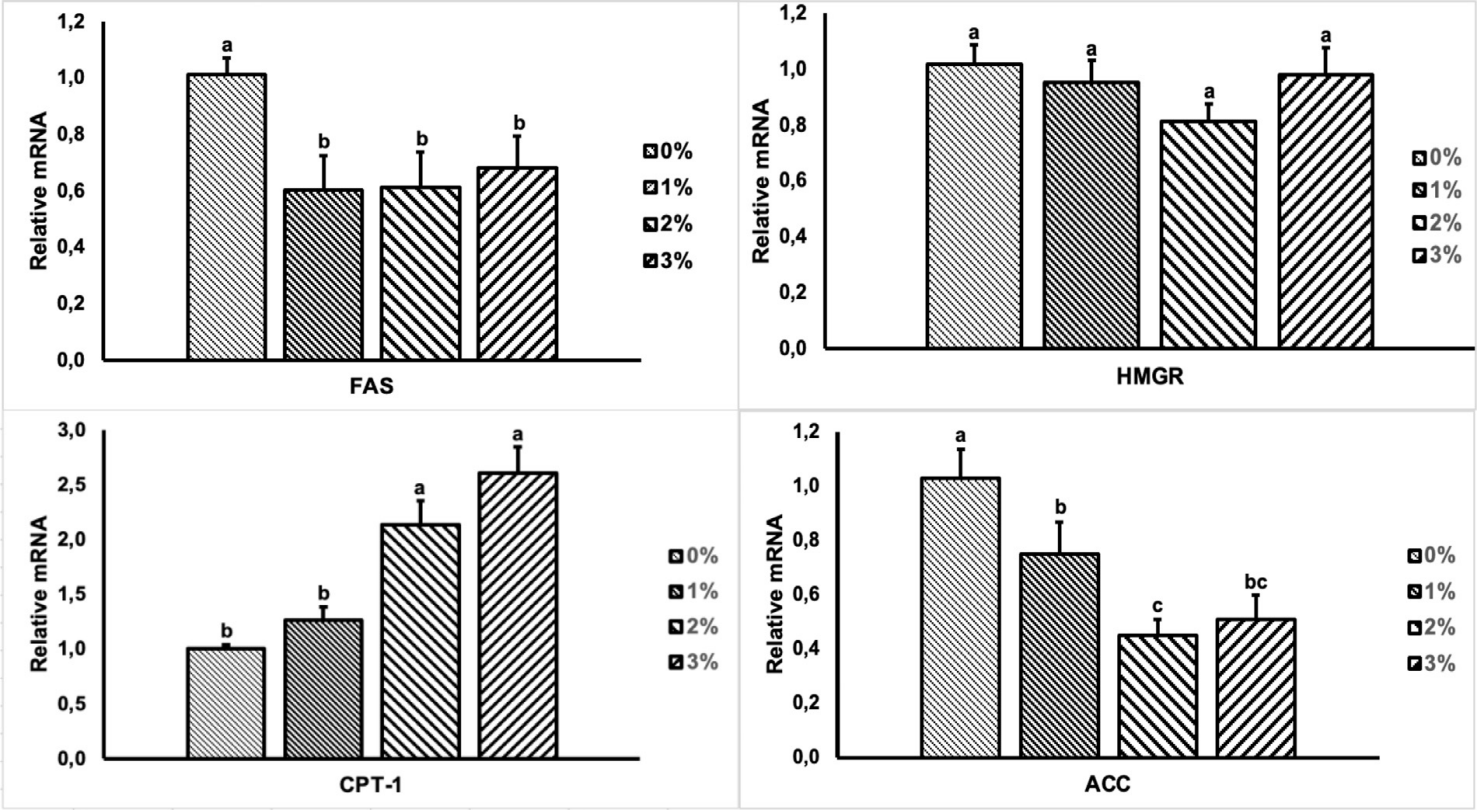
Comparison between FAS, HMGR, CPT-1, and ACC mRNA expression in broiler livers fed with the supplementation of black soldier fly larvae oil calcium salt (BSFLO-SCa). Data represent the mean value of 7 replicates with 1 bird per replicate. ^a–c^Different superscripts on the same target gene showed significant differences (*P* < 0.05).

Gene expression related to liver lipid metabolism in dietary supplementation of BSFLO-Sca is presented in Figure 1. Dietary supplementation significantly downregulated (*P* < 0.05) lipid synthesis gene expression (*FAS, ACC*). Dietary supplementation with 2 and 3% significantly upregulated (*P* < 0.05) fatty acid oxidation gene expression (*CPT-1*). There was no significant difference (*P* > 0.05) on cholesterol synthesis gene expression (*HMGR*).

## DISCUSSION

In the current study, dietary supplementation with BSFLO-SCa starting from 2% decreased their performance in terms of final FI, BWG, BW, and IP in broilers during the whole feeding period (from 11 to 35 d). Different results were reported, dietary supplementation with BSFLO did not affect FI, BWG, BW, and FCR (Schiavone et al., 2017; Dabbou et al., 2021; Kim et al., 2022). This difference may be attributed to changes in feed intake. The current study has shown that BSFLO-Sca decreased feed intake. In the same line, Marono et al. (2017) illustrated that BSF-L oil reduced feed intake in layers at 24 to 45 wk of age. Additionally, Kierończyk et al. (2022) reported that utilization of BSF-L oil reduced feed intake in broilers at the age of 1 to 14 d but had no effect at the age of 15 to 35 d. Several factors can cause this decrease. First, BSFLO can affect the feed's color, smell, and aroma so that it can change the palatability of the feed. Marono et al. (2017) reported that BSFLO has a brown and darker that can change the taste and aroma of the feed. Chickens can distinguish between the colors of the feed, which will affect the response to the amount of feed intake. Second, changing liquid oil into flour (calcium salt form) can make the feed's texture dustier. Warner et al. (2015) reported that calcium soap from long-chain fatty acids could reduce the acceptability of feed for livestock. The smell and taste of calcium soap can reduce the palatability of the feed so that it can reduce feed intake. Aguzey et al. (2018) explained that the dusty texture of the feed irritates the nose and eyes of animals. Moreover, it has a sour taste impacting palatability and consequently decreased feed intake and performance. Khurshid et al. (2019) show that decreased feed intake leads to nutrient insufficiency needed by the body for livestock maintenance, reproduction, and growth. Nutrient deficiency in livestock can be measured by bad performance in FI, BWG, BW, and FCR. These findings reported that supplementation of BSFLO-SCa increased FCR, which is associated with decreased of FI and BWG with supplementation of BSFLO-SCa. These finding supported the observation of Çalik et al. (2019), who reported that calcium salt tallow (**CST**) increased FCR compared with control that caused by decreased of FI and BWG with the increased of level supplementation.

Carcass characteristics are critical indicators in poultry production to describe the amount of nutrients deposited in the body and to evaluate the measurements of meat production. The factors that impact in carcass characteristics are the size of the noncarcass components (head, feet, organs, intestine, feathers, and internal fat), and the body mass or weight (Bai et al., 2022b). In the current study, supplementation of BSFLO-Sca to the diet starting from 2% reduced the slaughter, carcass, breast, and thigh weight compared to the control group. However, there was no significant difference in carcass, breast, and thigh percentages among all treatments. These results disagree with Kim et al. (2020) and Dabbou et al. (2021), who reported that supplementation broiler fed with BSFLO had no impact on the carcass characteristics. This difference may be attributed to low live weight in this current study (Table 5). Park et al. (2021) stated that carcass production is closely related to live weight or slaughter weights. Broilers with low live weight will produce low carcass weight.

Abdominal fat is a crucial indicator for assessing the overall body fat composition in poultry, which grows faster compared to other adipose tissues. Excessive fat deposition in tissue is linked directly with poor energy efficiency, excess feed energy, and reduced carcass yield (Fouad and El-Senousey, 2014). In the current study, the abdominal fat was significantly decreased by dietary supplementation with BSFLO-Sca starting from 1%. In similar regard that BSF larva reduced abdominal fat in layer (Nilugonda et al., 2022) and broiler chicken (Hartinger et al., 2021). This decrease can be attributed to the high lauric acid content in BSFLO-SCa. Wang et al. (2015) reported that using of coconut oil as a lauric acid source can reduce broiler's abdominal fat content. Marten et al. (2006) explained that MCFA could induce a decrease in body fat which several mechanisms can explain. First, MCFA are more susceptible to β-oxidation than those deposited in adipose tissue. Second, MFCA affect a rise in utilization of energy obtained from fat. Third, MCFA decrease gene expression involved in adipogenesis. This study's gene expression changes (Figure 1) can be associated with decreased abdominal fat.

Serum lipid profiles, including TG, LDL-cholesterol, HDL-cholesterol, and total cholesterol, serve as markers for assessing lipid metabolism within the body (El-katcha et al., 2021). In the current study, the serum HDL-cholesterol level was significantly increased by dietary supplementation with BSFLO-Sca at 3%. HDL-cholesterol is a lipoprotein that maintains the balance of cholesterol in the cell. HDL is good cholesterol because carry out the remaining cholesterol from the peripheral to the liver that using as a precursor formation of bile salt and a steroid hormone (Attia et al., 2017). In agreement with these findings, the utilization of BSF larvae increased HDL-cholesterol was followed by decreased LDL-cholesterol and cholesterol total (Park et al., 2017) and BSFLO increased HDL-cholesterol, LDL-cholesterol, and cholesterol total (Sypniewski et al., 2020). This result can be attributed to the high lauric acid content in BSFLO-SCa. The utilization of MCFA resulted in enhanced ApoA1 secretion, which was correlated with a rise in HDL-concentration (Shokrollahi et al., 2014). Apolipoprotein A1 (**ApoA1**) constitutes the predominant protein component of HDL particles. ApoA-I plays a significant role in the formation of HDL-C. The liver is the primary organ accountable for the synthesis and excretion of ApoA-I, with 70% of its production originating from this organ. Elevated levels of ApoA-I can lead to an enhancement in HDL levels within the bloodstream (Popeijus et al., 2021). In the current study, BSFLO-Sca increased serum albumin and total protein in broiler at 2 and 3%. In the same line, the concentration of serum albumin and total protein of broiler increased with supplementation BSF larvae (El-Kaiaty et al., 2022) and BSFL oil (Chen et al., 2022). MCFA are more ketogenic than LCFA so they can increase protein synthesis in the body. In addition, MCFA provide sufficient energy supply thereby reducing the utilization of protein as an energy source which results in an increase in protein concentration (Li et al., 2015).

Meat qualities become a major goal of the poultry industries that determined the economic and nutritional values of the meat products (Yao et al., 2023). There are 3 indicators in determine the quality of meat such as biological, physical and chemical quality (Mhlongo and Mnisi, 2023). In the current study, dietary supplementation with BSFLO-Sca decreased meat fat and cholesterol, while protein was increased. These finding supported the observation of Dabbou et al. (2021), who reported that using BSFLO decreased meat fat and Cullere et al. (2019) reported decreased meat cholesterol. The decreased in meat fat is associated with downregulated in the expression of genes involved in liver lipid synthesis (FAS, ACC), and upregulated in the β-oxidation (CPT-1) (Figure 1). Li et al. (2016) stated that supplementation of BSF larvae (high content of MCFA) decreased mRNA relative expression of FAS. Moreover, Wang et al. (2016) reported that MCFA supplementation such as caprylic and capric decreased in FAS, ACC, and SCD-1 expression. Mirghelenj et al. (2016) reported that a reduction in fat synthesis and an increase in the utilization of energy obtained from fatty acids can lead to a decrease in fat deposition within the body. The decreased meat cholesterol in the current study is associated with high levels of linoleic and oleic fatty acids in BSFLO (Table 1). Omega-3 fatty acids such as linoleic can suppress lipoprotein production or increase the clearance of circulating lipoprotein. On the other hand, omega-3 fatty acids have a role in reducing the expression of SREBP-1 (sterol regulatory element binding proteins), which reduces the enzyme expression involved in cholesterol synthesis (Abdulla et al., 2019). These findings reported that supplementation of BSFLO-SCa increased meat protein. Li et al. (2015) stated that MCFA increased blood protein content resulting from protein metabolism in the body which will be used for the formation of protein in meat. An increase in the protein content of meat is reflected by an increase in the protein content in the blood. These result accordance with the result of blood protein in this study (Table 7).

Physical quality, including pH value, color, cooking loss, and shear force, was used to evaluate sensory criteria of meat quality that affect the willingness of consumers to purchase meat products (Xie et al., 2022). Physical quality of breast meat, such as WHC, cooking loss, and color (a*, b*), was affected by dietary supplementation of BSFLO-Sca. In this study, the WHC increased. Contrary, the cooking loss decreased with the supplementation of BSFLO-Sca to the diet. In contrast to our findings, no apparent effect of dietary BSFLO on cooking loss and WHC is seen (Cullere et al., 2016; Gasco et al., 2019; Kim et al., 2020). The increase of meat protein affected the cooking loss and WHC (Table 8). Meat protein has a role in the binding of meat water. High protein content causes an increase in the ability to hold water so reduce the water loss during the cooking process (cooking loss) (Cabrol et al., 2022). The cooking loss value is closely related to the WHC of meat, whereas a low cooking loss follows a high WHC (Hutabarat et al., 2021). The WHC and cooking loss are indicators of meat products’ functional properties and process suitability that improve the tenderness and juiciness of the meat. Besides that, it also describes the amount of water loss and water-soluble nutrients (Park et al., 2021; Mahmoudi et al., 2022). In addition, a* (redness) and b* (yellowness) values increased with dietary supplementation of BSFLO-Sca. In agreement with these findings, dietary BSFLO increased the L*, a*, and b* values of the breast meat broiler (Schiavone et al., 2019; Kierończyk et al., 2023). Increased breast meat color results from an accumulation of xanthophyll pigments from BSF larvae. Sources of xanthophyll pigments come from vegetables and fruits with a high xanthophyll content as a substrate of BSF larvae (Schiavone et al., 2019; Heuel et al., 2022). The decrease of cooking loss in this study results in an enlargement of the muscle fibers and leading to a greater absorption of light by the meat surface, so the meat color looks redder (Hughes et al., 2014).

Meat fatty acid profiles of broilers are influenced by the fatty acid content of the diet (Skřivan et al., 2018; Saleh et al., 2021; Verge-Mèrida et al., 2022). Based on this, many studies have been done to modify the fatty acid composition of meat from the diet to produce bioactive fatty acids in animal products that are healthier for human nutrition (Abdulla et al., 2016; Long et al., 2020). The MCFA content in meat is a concern because it has an effect as an antiobesity, reduces body fat content in humans (Chu and Chiang, 2017), cardioprotective, antidiabetic, and antithrombotic (Choe et al., 2017). In the present study, supplementation of BSFLO-SCa affected the FA profiles of broiler. The available literature observed the same in broiler (Cullere et al., 2019; Kim et al., 2020; Kierończyk et al., 2023). BSFLO-SCa significantly increased MCFA (lauric and myristic) and MUFA in meat. Contrarily, concentration of PUFA decreased with supplementation of BSFL-SCa in line with the study Kim et al. (2020). Our study revealed that MCFA could be successfully incorporated into broiler meat by using BSFL-SCa. In addition, this study also found broilers with dietary supplementation of BSFLO-SCa had increased concentrations of oleic (C18:1 ω9), eicosanoic (C20:1 ω6), and eicosatetraenoic (C20:1 ω3) as well as decreased concentration of linoleic (C18:2 ω6) and nervoic (C24:1 ω3). Similarly, Cullere et al. (2019) found supplementation of BSFL oil in chicken diets had enhanced n-6/n-3 content in the meat of broilers. These results are likely due to the increased concentration of n-6 PUFA and decreased n-3 PUFA. A balanced ratio between n-6/n-3 can reduce fat deposition in the body by inhibiting fat synthesis (Yu et al., 2016), and the index may be linked with atherosclerosis and coronary heart disease (Milićević et al., 2014).

The liver is the leading site for lipid and cholesterol metabolism in poultry. The metabolism determines the amount of lipid and cholesterol accumulation in body tissues (Tian et al., 2019). Enzymes regulate this process at each reaction step, which is influenced by gene expression (Dev et al., 2021). In the production of lipid, ACC and FAS is the rate-limiting enzyme (Chen et al., 2018), while CPT-1 in the lipid oxidation (Lu et al., 2014), and HMGR in the cholesterol production (Eskandani et al., 2022). We evaluated the expression of ACC, FAS, CPT-1, and HMGR in the liver to investigate the influence of BSFLO-SCa on the molecular mechanism of lipid and cholesterol metabolism. The finding revealed that supplementation of BSFLO-SCa downregulated the expression of ACC and FAS while the expression of CPT-1 was upregulated. In the same line, in the recent study reported that utilization of MCFA, such as caprylic acid (C:8) and capric acid (C:10) downregulated the expression of FAS (Wang et al., 2015) and lauric acid (C12:0) from coconut oil downregulated the expression of ACC expression and upregulated the expression of CPT-1 (Arunima and Rajamohan, 2014). BSFLO-SCa as a MCFA source would help to inhibit hepatic FAS and ACC activity while increase CPT-1 activity. MCFA are known to be natural ligands that has effect on several transcription factors, such as downregulated the expression of sterol regulatory element binding proteins-1 (**SREBP-1**) resulting in decreased mRNA expression of genes involved in lipid production such as FAS and ACC (Eberlé et al., 2004) and upregulated the expression of peroxisome proliferator-activated receptors-α (**PPARα**) resulting in increased mRNA expression of genes involved in fat oxidation such as CPT-1 (Arunima and Rajamohan, 2014). Transcription factors bind to specific DNA sequences called enhancers or promoters to effect the conversion of gene information into a protein (Weidemüller et al., 2021). Fatty acids regulate the abundance of nuclear receptors on SREBPs while binding peroxisome proliferator response elements (**PPRE**) as part of DNA or directly binding to transcription factors on PPARs (Jump, 2004). In the current study, supplementation of BSL-SCa did not affect the expression of HMGR. In agreement with these findings, caprylic and capric acid did not affect the expression of HMGR. The decreased in meat cholesterol in this study (Table 8) because MCFA increased the gene expression involved in eliminating cholesterol (CYP7A1) (Xu et al., 2013). CYP7A1 is a gene that breaks down cholesterol and changes cholesterol to 27-hydroxycholesterol before it becomes bile (Eskandani et al., 2022).

In conclusion, dietary supplementation with BSFLO-SCa can be given up to 1% with the effect of reducing meat fat content, abdominal fat, and gene expression on lipid synthesis but does not affect performance (FI, BW, BWG, FCR, and IP) and carcass characteristics (carcass weight and breast weight). In addition, MCFA (lauric and myristic acid) meat content increased. The addition of BSFL-Sca of 2% or more decreased performance, carcass characteristics, and meat cholesterol and can improve meat protein and the physical quality of meat (WHC, a* and b*) and gene expression on fat oxidation, namely CPT-1.
